# *Clostridium difficile* infections in China^☆^

**DOI:** 10.1016/S1674-8301(10)60055-3

**Published:** 2010-11

**Authors:** Ke Jin, Shixia Wang, Zuhu Huang, Shan Lu

**Affiliations:** a Department of Infectious Diseases,; b Jiangsu Province Key Laboratory in Infectious Diseases,; c China-US Vaccine Research Center, the First Affiliated Hospital of Nanjing Medical University, Nanjing, Jiangsu 210029, China;; d Department of Medicine, University of Massachusetts Medical School, Worcester, MA 01655, USA.

**Keywords:** *Clostridium difficile*, review, prevalence, incidence, risk factors

## Abstract

*Clostridium difficile* (*C. difficile*) infection has become one of the major hospital-associated infections in Western countries in the last two decades. However, there is limited information on the status of *C. difficile* infection in Chinese healthcare settings. Given the large and increasing elderly population and the well-recognized problem of over-prescribing of broad spectrum antibiotics in China, it is critical to understand the epidemiology and potential risk factors that may contribute to *C. difficile* infection in China. A literature review of available published studies, including those in Chinese language-based journals, was conducted. A review of the currently available literature suggested the presence of *C. difficile* infections in China, but also suggested that these infections were not particularly endemic. This finding should lead to better designed and greatly expanded studies to provide a more reliable epidemiologically-based conclusion on the actual status of *C. difficile* infection in China, including the identification of any associated risk factors. Such information is ultimately valuable to develop appropriate strategies to prevent *C. difficile* infection and the vast negative impact of such infections in China and other developing countries.

## INTRODUCTION

*Clostridium difficile* (*C.difficile*) is an anaerobic Gram-positive bacillus that possesses the ability to form spores resistant to many commonly used hospital disinfectants and can survive in the hospital setting more than six months[1]. Therefore it can be widespread in the hospitals through medical devices, floors and hands of medical staff[2],[3]. *C. difficile* is one of the major emerging hospital infections in Western countries and its prevalence is increasing at an alarming rate in recent years. However, the epidemiology of *C. difficile* infections in developing countries, including China, is unclear and is further complicated by the limited number of publications focused on *C. difficile* infections in China in the English-based literature. At the same time, the awareness of *C. difficile* infections among general healthcare providers in China is poor and only a small subset of infectious disease specialists understand that *C. difficile* infections should be included in the routine differential diagnoses for hospitalized patients presenting with diarrhea. This review summarizes the information related to *C. difficile* infections in China from both English-based and domestic medical literatures, and should provide a current reference to worldwide healthcare providers, epidemiologists, health policy decision makers and developers of novel anti-*C. difficile* treatments for their potential interest in understanding and managing the global trend of *C. difficile* infections.

## INCIDENCE AND DISEASE TRENDS

It is well documented that the incidence of *C. difficile* infections is rising in Western countries over the past 1-2 decades. In 1996, the rate of *C. difficile* infections in the United States hospitals was 31 cases/100,000 patients; by 2003, this rate soared to 61 cases/100,000 patients[4]. The study by the Association for Professionals in Infection Control and Epidemiology showed that the detection rate of *C. difficile* was 13.1‰ and the incidence was 12.4‰ in hospitalized patients, a significant increase over the previous data[5]. According to one study, in short-stay hospitals in the United States, the number of discharges for which *C. difficile*-associated disease was listed as a diagnosis more than doubled from 82,000 (31/100,000 population) in 1996 to 178,000 (61/100,000 population) in 2003[6] and in another study in the United States, from 2000-2006, there was a significant increase in the overall incidence rate of healthcare facility onset, and healthcare facility-associated *C. difficile* infections (7.0-8.5 cases per 100,000 patient days) each year[7]. For comparison purposes, a total of 6,201 episodes of *C. difficile* infections were identified (118.3/100,000 population)[8] and in the Netherlands, in 2008, the incidence of *C. difficile* infection was 18.0 per 100,000 admissions from 14 participating hospitals[9]. Furthermore, since Canada first reported a highly virulent *C. difficile* NAP-1/027, this strain has spread to the USA, Europe and Asia, and caused local outbreaks[10]-[14]. Overexpression of exotoxins and resistance to quinolones by NAP-1/027 increased *C. difficile* infection-related mortality rate to 6.9%, a highly alarming level[11],[15]. Based on a review of the global epidemiology of *C. difficile* infections throughout the world, this strain has been largely responsible for increases in *C. difficile* infection rates in North America and Europe[16].

At the same time, there is a lack of published reports on the overall incidence of *C. difficile* infections at the national level in China. In one study, conducted for a one-year period (2007-2008) at Huashan Hospital, a major teaching hospital in Shanghai, China, stool samples of 587 suspected cases of *C. difficile* infections were examined[4],[5],[17]-[20]. Seventy-four patients (12.6%) tested positive for *C. difficile* by bacterial culture and among these, up to 13 (17.6%) were toxin negative[20]. Among the general inpatient population in southern China, 183 patients with diarrhea from the First Affiliated Hospital of Zhongshan Medical University in Guangzhou were identified between 1994 and 1997[10]. Among these 183 patients, 21 (11.5%) were diagnosed with *C. difficile* infections. A second study in southern China showed that, among 257 hospitalized patients with a diagnosis of diarrhea caused by the use of antibiotics, 5.06% were tested positive for *C. difficile*[21]. In a study conducted in northern China at Beijing Hospital, 36 cases of *C. difficile* infection were identified among a total of 71,428 inpatients from 1998 to 2001[11].

The incidence of *C. difficile* infections has also been studied among specific inpatient populations since this type of infection is quite prevalent in patients with malignancy. Among a group of patients receiving routine chemotherapy in the Inner Mongolia region (*N* = 2,778), 44 patients (1.6%) had *C. difficile* infection[13]. However, in another study conducted in the same region, among 1,074 patients receiving chemotherapy, 38 patients (3.5%) were diagnosed with *C. difficile* infection[14]. One study of 4,697 patients receiving chemotherapy in Tianjin identified 87 patients (1.9%) who were positive for *C. difficile*[15]. For other inpatient populations, one study reported an incidence rate of 3.57% among 140 patients in an inpatient neurology ward during a 3-month period in 1998-1999[22]. In another study, two cases of *C. difficile* infection were reported among 36 patients with diarrhea associated with antibiotic use from post-operative neurosurgery[23]. In a population of 44 patients with diarrhea who received allogeneic hematopoietic stem cell transplantation, 12 cases (27.27%) were diagnosed with *C. difficile* infection[24]. In a pediatric population, *C. difficile* infection rate was observed to be 1.59% (11/693) in patients with persistent and chronic diarrheal disease and 0% in patients with acute diarrheal disease in one study[25].

Several factors may influence the incidence of *C. difficile* infection rates in hospitalized patient populations in China although none has been systematically determined. It was the opinion of local physicians in China that the overall clinical condition of hospitalized patients in China is less severe than those admitted in Western countries; there is a lack of clear guidelines in Chinese hospitals for sending stool samples from patients with diarrhea for laboratory testing, and clinicians may begin treatment without laboratory confirmation of diagnosis. Similar to that in Western countries, a diagnosis of *C. difficile* infection, using traditional bacterial culture methods, is not consistently conducted in many hospitals and newer toxin-based diagnostics are not available in routine clinical practice due to the high cost of such diagnostic kits.

## C. DIFFICILE RELAPSE RATE IN CHINA

One important parameter for *C. difficile* control is to prevent recurrent infection in the same patient population. Currently, there is a lack of reliable information on the relapse rate of *C. difficile* infections. In one study conducted at the Huashan Hospital, 5 (8.9%) recurrent cases among 56 confirmed *C. difficile* infections were observed during a one-year study period[20]. In this study, recurrences were defined as patients with the reappearance of symptoms and positive culture at least 7 days but less than 30 days after resolution of the previous diarrheal episodes and discontinuation of antimicrobial therapy. It was also observed that all isolates from the recurrent episode were similar in that they shared the same toxin profile and same PCR ribotype as the isolates from the initial episode. However, among patients receiving allogeneic hematopoietic stem cell transplantation, a high relapse rate (16.67%) was observed even when proper antibiotic treatment was employed[24].

## SEVERITY OF *C. DIFFICILE* DISEASE IN CHINA

Since no clear definition of severity has been associated with *C. difficile* infection in Chinese-based hospitals, an inconsistent description of infection severity often occurs and many studies often fail to describe severity associated with this infection. In one extreme report among 140 patients in the neurological ward, five cases (3.57%) were diagnosed with *C. difficile* infection. Four cases were described as severe or worse and only one case was classified as mild[22]. Two out of these five patients died as a result of complications associated with severe *C. difficile* infection.

## RISK FACTORS ASSOCIATED WITH *C. DIFFICILE* INFECTIONS IN CHINA

Underlying co-morbidity has been identified as a possible risk factor in *C. difficile* infections. Malignancy appears to contribute to an increased rate of *C. difficile* infections when compared to the general hospitalized population. In one study, among 21 patients with *C. difficile* infection in southern China, malignancy accounted for 10 cases (47.6%) compared with *C. difficile* infection in patients receiving surgery (8 cases; 38.1%) and chronic diseases of major organs (3 cases; 14%)[10]. Poor health due to chronic illness appeared as another key factor in *C. difficile* infection rates. In a study conducted in northern China, out of 36 cases of *C. difficile* infection, 28 patients (77.8%) presented with severe chronic illness[26] and in another study, 30 out of 44 cases with *C. difficile* infection also suffered from chronic diseases[27].

Another risk factor associated with increased *C. difficile* infection is the use of broad spectrum antibiotics, especially cephalosporins, broad-spectrum penicillin, quinolones and aminoglycosides[10],[11],[28]. In China, antibiotics are readily available and are often over-prescribed; it is clear from most studies that use of more than one type of antibiotic may lead to *C. difficile* infection as part of the phenomenon called antibiotic associated diarrhea (AAD). It is hypothesized that the inappropriate use of antibiotics, especially the broad-spectrum antibiotics, causes a significant reduction of naturally-occurring gut flora, allowing opportunistic pathogens to colonize and possibly cause disease. *C. difficile*, being one of these opportunistic pathogens, secretes exotoxins. These exotoxins, when accompanied by the loss of gut flora and their ability to degrade these toxins, allows *C. difficile* toxins to bind intestinal epithelial cells and activate the downstream signaling pathway, leading to the aggregation of inflammatory cells and the massive release of inflammatory mediators. This cascade of events eventually results in edema or necrosis of the intestinal tract, which is presented as abdominal pain and diarrhea in the patient.

The immune status of the host may also contribute to increased *C. difficile* infection rates as was observed in patients receiving chemotherapy or allogeneic hematopoietic stem cell transplantation[13]-[15],[24]. However, these results are also confounded by the fact that many of these patients are also receiving broad spectrum antibiotics at the time of *C. difficile* infection.

Other risk factors associated with increased *C. difficile* infections were identified as having residence in a nosocomial setting and the use of respiratory intubation and stomach intubation[29] as these risk factors were associated with a high level of C reactive protein (CRP) and low level of albumin[27]. However, there is a lack of strong clinical data to definitively support these relationships.

## ENDEMIC *C. DIFFICILE* STRAINS IN CHINA

In one study conducted in hospitalized patients, among 56 isolates of *C. difficile* that were identified positive for toxins, 43 (77%) were positive for both toxin A and toxin B, 13 (23%) were negative for toxin A and positive for toxin B[20]. Furthermore, neither binary toxin nor TcdC deletion was identified in any of the isolates. As for the drug resistance of these 56 *C. difficile* isolates, resistance to moxifloxacin, ciprofloxacin, levofloxacin, erythromycin, clindamycin, tetracycline and rifampicin was found in 46.4%, 100%, 60.7%, 71.4%, 71.4%, 35.7% and 25.0% of the isolates, respectively, and all strains were susceptible to metronidazole, vancomycin, meropenem and piperacillin/tazobactam. Fourteen different ribotypes were identified, a specific clone, SH II, accounted for 25% of isolates whereas no isolates belonged to ribotype 027. In a more recent study reported in two related manuscripts, 12 cases (10.7%) of *C. difficile* infection were identified from 112 patients with diarrhea in a general hospital in Beijing[30],[31]. Among these 12 patients, 8 (66.7%) were toxin positive; five were positive for both toxin A and toxin B while three were negative for toxin A but positive for toxin B. Various levels of drug resistance of eight toxic positive *C. difficile* isolates have been identified[32]. Thirty-seven percent were identified as resistant to ampicillin, 87.5% to clindamycin, and 12.5% to metronidazole; however, none of the isolates from this study was resistant to vancomycin. These eight isolates were mapped onto four gene types with ZR I as the dominant type (62.5%)[30],[32].

## TREATMENT OF *C. DIFFICILE* INFECTION

Discontinuation or a change in antibiotics, proton pump inhibitor (PPI) and other agents may be enough to rid of *C. difficile* associated diarrhea. In one study, Chen *et al.*[10] documented that in 100% of patients (11/11) discontinuation of antibiotics eliminated mild *C. difficile* infection. Since the number of *Lactobacilllus, Bifidobacteria*, and other naturally-occurring gut bacteria decreased significantly in patients with *C. difficile* infection[24],[33], prescription of probiotics such as *Live Bacillus Licheniformis* preparation Dral*, Bifid Lriple Viable*, and *Lostridium Butyricum*, *Miyarisan* may be beneficial[14],[24]. In one study, in all 10 symptomatic patients included in the study, pseudomembranous colitis disappeared after the use of *Live Bacillus Licheniformis* preparation Dral[34]. In moderate or severe cases, gamma globulin, which may contain specific antibodies against toxins of *C. difficile,* has been prescribed[35]. However, the most commonly used treatment was the addition of metronidazole due to its ability to inhibit bacterial DNA synthesis, and because it is easily affordable[36]. However, due to gastrointestinal side effects, increasing drug resistance rate, and relative inefficiency of metronidazole, local physicians in China typically prefer vancomycin over metronidazole, considering the fast-acting, high-efficacy, low recurrence and resistance rates of vancomycin[10],[13]. In one study, among 102 patients who developed diarrhea after receiving chemotherapy, 87 cases were positive for *C. difficile* and all were effectively treated with vancomycin[15].

Chinese herbal medicine has commonly been used in clinical practice for *C. difficile* infection in China. One report treated 72 cases with antibiotic-associated colitis using a *Puerariae Radix, Scutellariae Radix*, and *Rhizoma Coptidis* decoction and showed that the disappearance of symptoms occurred faster in those who received the herbal remedy compared to control[26]. In another study, total effective rate in the group that received Four Miraculous Drugs plus vancomycin was 93.33% while the total effective rate was 76.66% in the vancomycin alone group[37]. Dai *et al.* also reached the same conclusion when using garlic preparations in the treatment of pseudomembranous colitis[38].

## CONCLUSION

The actual incidence of *C. difficile* infections in China is not known due to a lack of large-scale studies; however, easy to use and inexpensive diagnostic tests are needed for such studies. Some of the limited studies conducted so far suggest that the *C. difficile* infection rate in the general in-patient population may be lower than rates reported in the Western countries in recent years. However, *C. difficile* infection in high risk patients such as those in ICU and oncology service may be more prevalent. For *C. difficile* infections confirmed with laboratory tests, most are dual positive for both toxin A and toxin B, and positivity for only one toxin (toxin B) was also observed. No new toxin subtype has been identified. The antibiotic treatment for *C. difficile* infection is similar to what is used in the Western countries. Use of alternative approaches including herbal medications is reported in selected studies.

## References

[1] Vonberg RP, Kuijper EJ, Wilcox MH, Barbut F, Tull P, Gastmeier P (2008). Infection control measures to limit the spread of Clostridium difficile. Clin Microbiol Infect.

[2] Mutters R, Nonnenmacher C, Susin C, Albrecht U, Kropatsch R, Schumacher S (2009). Quantitative detection of Clostridium difficile in hospital environmental samples by real-time polymerase chain reaction. J Hosp Infect.

[3] Dubberke ER, Reske KA, Noble-Wang J, Thompson A, Killgore G, Mayfield J (2007). Prevalence of Clostridium difficile environmental contamination and strain variability in multiple health care facilities. Am J Infect Control.

[4] Huang H, Wu S, Wang M, Zhang Y, Fang H, Palmgren AC (2008). Molecular and clinical characteristics of Clostridium difficile infection in a University Hospital in Shanghai, China. Clin Infect Dis.

[5] Huang H, Weintraub A, Fang H, Nord CE (2009). Antimicrobial resistance in Clostridium difficile. Int J Antimicrob Agents.

[6] McDonald LC, Owings M, Jernigan DB (2006). Clostridium difficile infection in patients discharged from US short-stay hospitals, 1996-2003. Emerg Infect Dis.

[7] Dubberke ER, Butler AM, Yokoe DS, Mayer J, Hota B, Mangino JE (2010). Multicenter study of Clostridium difficile infection rates from 2000 to 2006. Infect Control Hosp Epidemiol.

[8] Kotila S, Virolainen A, Snellman M, Ibrahem S, Jalava J, O (2010). L. Incidence, case fatality and genotypes causing Clostridium difficile infections, Finland, 2008.. Clin Microbiol Infect.

[9] Hensgens M, Goorhuis A, Notermans D, van Benthem B, Kuijper E (2010). Changing epidemiology of infections in the Netherlands in 2008/'09. Ned Tijdschr Geneeskd.

[10] Chen DM, Chen QG, Chen JX (2000). 21 cases of Clostridium difficile colitis. Guangdong Medical J (in Chinese).

[11] Wang W, Liu X (2004). Clinical analysis of 36 cases of Clostridium difficile colitis. Chin J Medicine (in Chinese).

[12] Yang YH, Zhang CP, Cai H (2006). Investigation on the causes of intestinal bacterial flora imbalance and treatment of drug-resistant. Chin J Nosocomiol (in Chinese).

[13] Du RL (2007). Clinical study on chemotherapy-induced diarrhea. Inner Mongolia Med J (in Chinese).

[14] Chen C, Qiu L (2006). The study enteroflora and treatment of digestive tracty chemotherapy-induced diarrhea. Chin J Clin Gastroenterol (in Chinese).

[15] Jiang Z, Qiu L, Zhu X (2003). Clinical research on norvancomycin treatment of chemotherapy-related diarrhea. Chin J Intern Med (in Chinese).

[16] Gerding DN (2010). Global epidemiology of Clostridium difficile infection in 2010. Infect Control Hosp Epidemiol.

[17] Huang H, Fang H, Weintraub A, Nord CE (2009). Distinct ribotypes and rates of antimicrobial drug resistance in Clostridium difficile from Shanghai and Stockholm. Clin Microbiol Infect.

[18] Huang H, Weintraub A, Fang H, Nord CE (2009). Comparison of a commercial multiplex real-time PCR to the cell cytotoxicity neutralization assay for diagnosis of clostridium difficile infections. J Clin Microbiol.

[19] Huang H, Weintraub A, Fang H, Nord CE (2009). Community acquired Clostridium difficile infection due to a moxifloxacin susceptible ribotype 027 strain. Scand J Infect Dis.

[20] Huang H, Wu S, Wang M, Zhang Y, Fang H, Palmgren AC (2009). Clostridium difficile infections in a Shanghai hospital: antimicrobial resistance, toxin profiles and ribotypes. Int J Antimicrob Agents.

[21] Yang Y, Zhang J, Cai H (2006). Intestinal tract dysbacteriosis: causes, drug resistance and treatment investigation. Chin J Nosocomiol (in Chinese).

[22] Chen W, Jin G, Liu Y (2001). Report of Clostridium difficile infection in elderly patients in neurological department. Military Med J South China (in Chinese).

[23] Liu X, Song J, Zhang X (2006). Antibiotic-associated diarrhea in post-operative neurosurgical patients: clinical features, diagnosis and treatment of 36 cases. Chin J Mod Med (in Chinese).

[24] Jia JS, Huang XJ, Liu DH, Xiu LP, Zhang YC, Wu T (2008). Relationship between Clostridium difficile associated diarrhea and intestinal microecosystem disorder in patients received allogeneic hematopoietic stem cell transplantation. J Exp Hematol.

[25] Cai X, Lu D, SQ C (2001). Changes of pathogens and antibiotic sensitivity in children with persistent and chronic diarrheal disease during a decade. Clin Med of China (in Chinese).

[26] Wang Y (2003). Integrated treatment of 72 cases with antibiotic-associated colitis. Chin J Integrative Med (in Chinese).

[27] Huang Y, Nie Y (2009). Clinic epidemic comparisons of Clostridium difficile-associated diarrhea with antibiotic-associated diarrhea. J Guangdong Med Coll (in Chinese).

[28] Kuijper E, Barbut F, Brazier J (2008). Update of Clostridium difficile infection due to PCR ribotype 027 in Europe, 2008. Euro Surveill.

[29] Chen W, Jin G, Liu YY (2001). Report on hospital outbreaks of Clostridium difficile infection in hospitalized elderly patients. Chin J Nosocomiol (in Chinese).

[30] Cheng Y, Lu J, Yan S (2009). Primary study on the gene typing, molecular characteristics of virulence and resistance associated gene of 12 Clostridium difficile clinical isolates in China. Chin J Zoonoses (in Chinese).

[31] Cheng Y, Lu JX, Yan SK, Jia HB, Li WG (2009). Study on toxin characteristics of Clostridium difficile isolated from hospital. Dis Surv (in Chinese).

[32] Cheng Y, Lu J, Yan S (2009). Study on drug resistance of Clostridium difficile isolated from hospital. Dis Surv (in Chinese).

[33] Cao Y, Zhang X, Pan LJ (2002). The clinical research between Clostridium difficile colonitis and intestinal microecosystem. Chin J Mod Med (in Chinese).

[34] Xu X, Yang Z (2001). Report on the treatment of Live Bacillus Licheniformis preparation Dral in 10 cases with pseudomembranous colitis after abdominal surgery. J Qiqihar Med Coll (in Chinese).

[35] Wei H, Wang K (2002). Observation of high-dose intravenous gamma globulin treatment in children with antibiotic associated diarrhea. Med J Qilu (in Chinese).

[36] Luan X, Wang J, Yang X (2004). Clinical analysis in 28 patients with clostridium difficile colitis. J Taishan Med Coll (in Chinese).

[37] He S, Yuan H (2006). Integrated treatment of 60 cases with Pseudomembranous colitis. Guangxi J Trad Chin Med (in Chinese).

[38] Dai T (2010). Garlic preparations as an adjuvant of antipyretic dryingdampness agents for the treatment of Pseudomembranous Colitis. J Hubei Univ Chin Med (in Chinese).

